# Atypical Presentation of Fatal Disease: Painless Aortic Dissection

**DOI:** 10.7759/cureus.14355

**Published:** 2021-04-07

**Authors:** Genanew Bedanie, Fuad I Abaleka, Alay Tikue, Thanita Thongtan, Mohammad M Ansari

**Affiliations:** 1 Internal Medicine, Texas Tech University Health Sciences Center, Lubbock, USA; 2 Internal Medicine, Richmond University Medical Center, Staten Island, USA; 3 Cardiology, Texas Tech University Health Sciences Center, Lubbock, USA

**Keywords:** type b aortic dissection, atypical presentation of aortic dissection, aortic dissection, dizziness in aortic dissection

## Abstract

Aortic dissections (ADs) are uncommon but they are highly lethal. Due to atypical signs and symptoms, diagnosis of type B AD can be easily missed or delayed. Our patient presented to the emergency center with dizziness and fall for which he was evaluated and treated for hypertension. Two weeks later, he again presented to the hospital with painless right groin swelling: computed tomography (CT) of the abdomen incidentally showed type B AD. The patient might have an AD presenting with dizziness and hypertension during previous presentation. From this case, we learn how it is challenging to diagnose painless AD especially when patient comes with atypical symptoms. In high-risk patients with unexplained dizziness and fall, a high degree of suspicion may help for early diagnosis and management.

## Introduction

Aortic dissection (AD) is a life-threatening condition associated with significant mortality. Painless aortic dissection is relatively rare, and can be easily missed due to its atypical presentation. Many patients die before arriving at the hospital or prior to diagnosis [1]. The estimated incidence ranges from 2.6 to 3.5 cases per 100,000 person-years [2]. Classical symptoms of AD include an acute onset of severe chest, back, and abdominal pain characterized as tearing or ripping in nature. Patients with painless aortic dissection present with atypical symptoms with no chest pain. In at-risk patients, a high index of suspicion is required for early diagnosis and to improve survival. We will discuss a patient who presented with atypical symptoms due to type B aortic dissection.

## Case presentation

**Figure 1:**
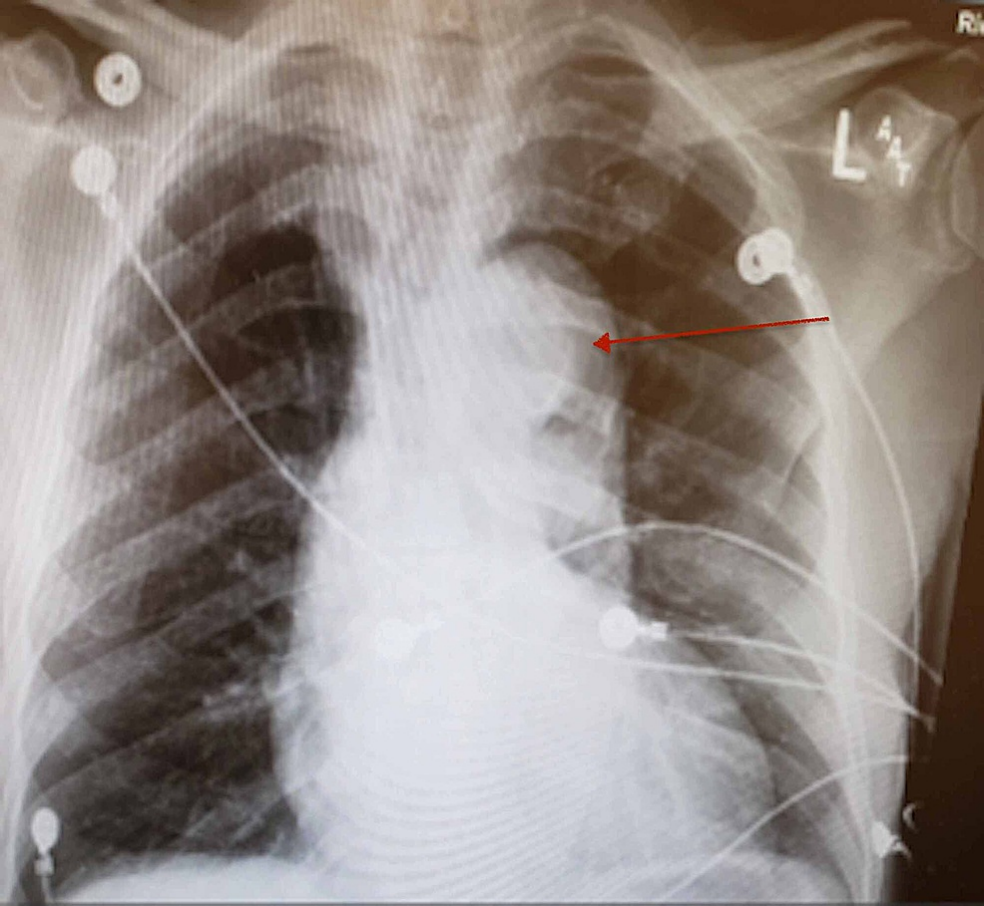
Chest x-ray: mediastinal widening with positive calcium sign (inward displacement of aortic wall calcification by more than 10 millimeters).

**Figure 2:**
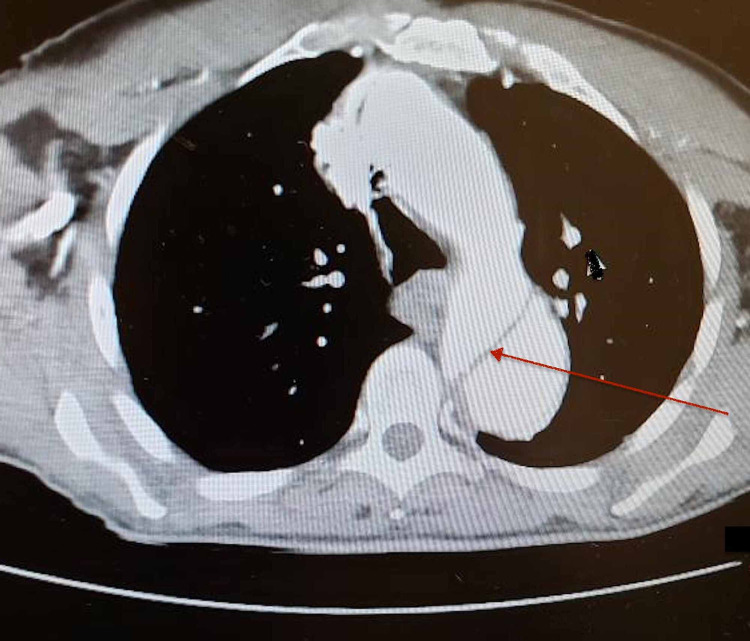
CT chest showing aortic dissection with an intimal flap.

**Figure 3:**
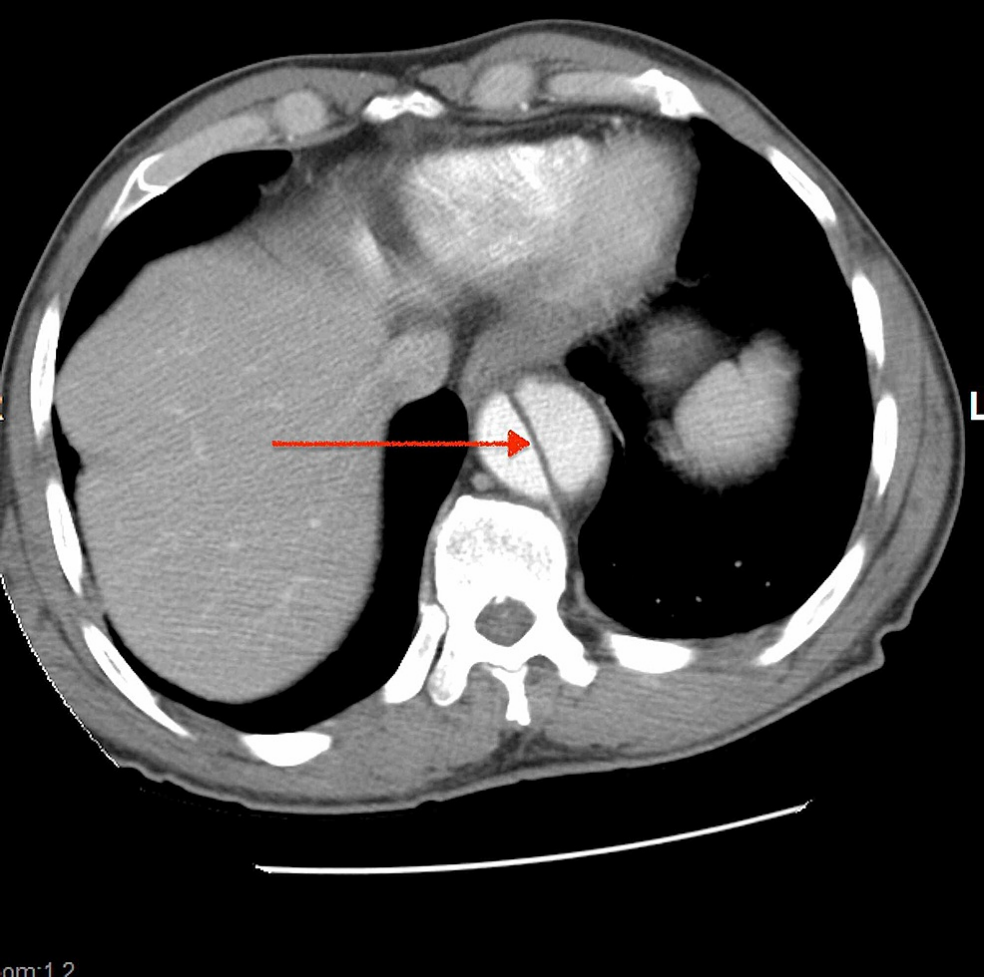
CT abdomen showing type B aortic dissection (supra renal) with true and false lumens separated by an intimal flap.

**Figure 4:**
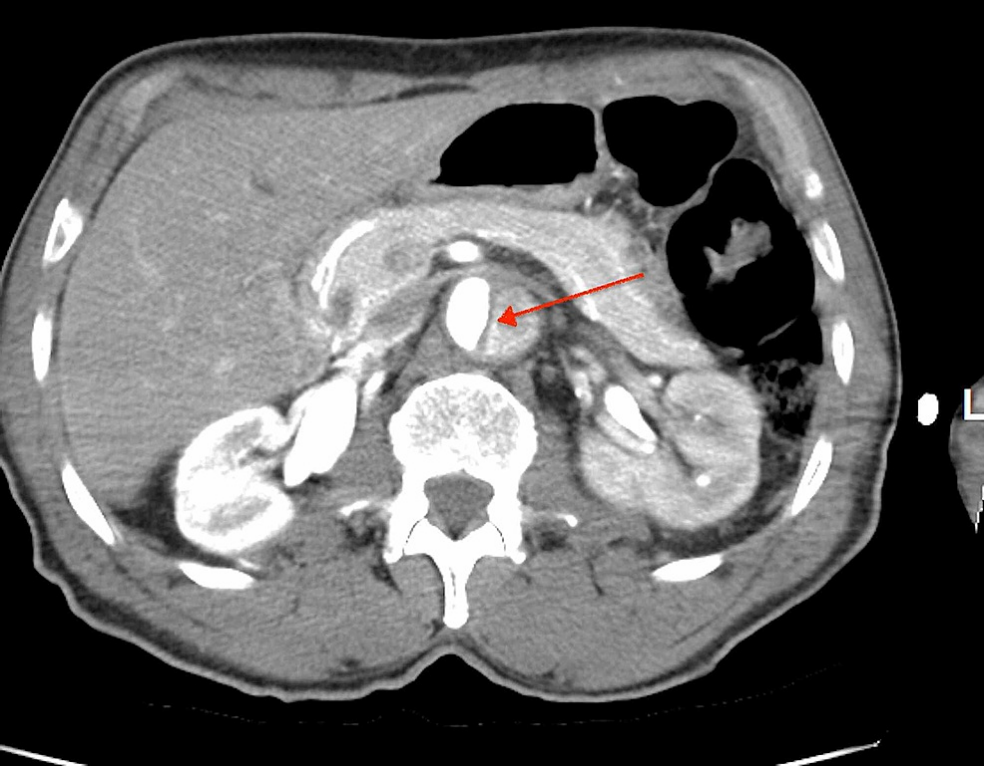
CT abdomen showing type B aortic dissection with true and false lumens separated by an intimal flap at the level of kidneys (renal arteries).

An 80-year-old man with hypertension presented to the emergency center after he noticed painless swelling over his right groin of two days duration. He also visited the emergency department two weeks earlier after an accidental fall caused a small facial laceration that was repaired with stitches. Patient had mild dizziness associated with the fall, which resolved spontaneously. Patient stated he was missing his antihypertensive medication occasionally. Otherwise he denied any chest, back, or abdominal pain. Patient has been smoking 5-6 cigarettes a day for more than 20 years. He denies any recreational drug use. Examination during previous emergency center visit showed blood pressure of 160/95 mmHg, heart rate of 76 beats/minute and respiratory rate of 12 breaths/minute. No other abnormal physical findings except laceration on forehead. Electrocardiogram (EKG) and CT head were normal. Forehead wound was sutured; patient was advised to take his antihypertensive medications. He was discharged home, and he was recommended to have follow-up with his primary care physician. During the second hospital visit, blood pressure was 184/94 mmHg, pulse rate 77 beats/minute; respiration rate 16 breaths/minute, body temperature 98.2^0^F, and oxygen saturation was 96% in room air. Patient was well-looking and not in pain. There was a healthy sutured wound on the forehead. Chest was clear bilaterally. Heart sounds were normal with no murmurs or gallop. Abdominal examination revealed a right reducible inguinal hernia, the abdomen was otherwise soft and nontender. There was no significant pulse deficit. Patient was alert and oriented with no neurological abnormalities. His laboratory results revealed: White blood cell count of 5,600/ul, hemoglobin 12.5 gm/dl, BUN 17 mg/dl, creatinine 1.3 mg/dl (baseline creatinine was 1.2 mg/dl). Urine studies were within normal limits and negative for drug screen. Transthoracic echocardiogram revealed left ventricular ejection fraction of 55%, thickening of aortic valve, and mild left ventricular hypertophy. Scrotal ultrasound showed minimal bilateral hydroceles and right inguinal hernia. Chest X-ray revealed mediastinal widening with positive calcium sign (see Figure 1). Abdominopelvic CT with contrast showed an incidental finding of aortic dissection and indirect right inguinal hernia. In order to further delineate the extent of the dissection, CT angiogram was done and it confirmed Type B aortic dissection that extending from the origin of right subclavian artery to the bifurcation of the common iliac arteries (see Figures 2-4). The patient was admitted to the medical intensive care unit for blood pressure control and close monitoring. Vascular surgery was consulted and recommended conservative medical treatment. He was managed conservatively with labetalol and IV nicardipine in the unit and subsequently transferred to the general medicine floor. Patient remained hemodynamically stable; his blood pressure was well controlled, and he was discharged in stable condition with an outpatient follow-up appointment. 

## Discussion

Acute AD is one of the life-threatening cardiovascular emergencies associated with significant mortality. According to the Stanford classification, AD is classified as type A and type B. This classification helps to separate type A which needs surgical management, whereas type B usually requires only medical management.

AD can present with a wide range of manifestations, and the classical clinical presentation may be absent [3]. 4.5%-6% of ADs are painless and the chances of missing the diagnosis are very high in those cases [4, 5]. Physicians need to be aware of these atypical presentations and should consider as a possible differential diagnosis in high-risk patients.

AD happens when an intimal tear leads to the leakage and propagation of blood through the aortic media creating a false lumen. The dissection may propagate proximally and can involve the heart or distally the descending aorta and its major branches. Pathogenesis is related to factors that contribute to aortic wall medial degeneration and those that increase aortic wall stress. Hypertension is the most significant risk factor for AD [5,6]. Other risk factors include age, smoking, dyslipidemia, cocaine use, hereditary disorders (Marfan syndrome, Ehlers-Danlos syndrome, Turner syndrome, bicuspid aortic valve, coarctation of the aorta, preexisting aortic aneurysm), vasculitis, trauma, and iatrogenic factors.

Hypertension was seen on initial presentation more commonly among patients with type B dissection (70.1% vs 35.7%, P<0.001) [5]. A study from a large referral center (which involved 236 cases) revealed the diagnosis of AD was missed in 38 % of patients on initial evaluation, and in 28 % of patients the diagnosis was established on postmortem examination [7].

A review of 977 patients in the International Registry of Acute Aortic Dissection (IRAD) database by Park et al. revealed about 63 (6.4%) patients had painless AD, and these patients were found to have a higher incidence of syncope, congestive heart failure and stroke relative to the classic painful AD patients. Mortality and aortic rupture were also higher among patients with painless Type B AD compared to Painful Type A AD [6]. Pape et al. analyzed the data of 4,428 patients recorded in the IRAD database over a period of 17 years, per their report the common presentations in painless dissection were syncope (33.9%), new-onset neurological deficit (23.7%), congestive heart failure (19.7%), coma or spinal cord ischemia (17.0%), acute renal failure (13.6%), myocardial infarction (7.1%), and mesenteric ischemia or infarction (6.8%) [8].

Diagnosis depends on clinical presentation and imaging. High index of suspicion is very important in at-risk patients. Chest X-ray may be normal or reveal nonspecific findings that can help in making decision for further workup. The IRAD study by Pape et al. revealed about 43.1% to 54.3% of patients had mediastinal widening, but this finding can go up to 85% of cases [8]. One-third of chest X-ray in AD are normal to the untrained eye. Loss of aortic knob and positive calcium sign (inward displacement of aortic wall calcification by more than 10 mm) are important findings in AD. Other X-ray findings include diffuse enlargement of the aorta, tracheal shift to the right, pleural effusion, and cardiac enlargement [9]

Magnetic resonance imaging (MRI), computed tomography (CT) or transesophageal echocardiography (TEE) are diagnostic with sensitivities of almost 98.3%, 98.3%, and 97.7%, respectively. In patients with unstable hemodynamic status, TEE is the preferred diagnostic modality. Transthoracic echocardiography (TTE) has only 59.3% sensitivity [10]. MRI, CT and TTE imaging usually reveal an intimal flap allowing rapid and accurate diagnosis of AD [11].

Treatment of AD depends on the type of dissection and associated complications. Type A AD usually requires emergent surgery. Whereas type B AD is usually treated medically, but it may need surgical or endovascular intervention when it is associated with complications like aortic expansion, progression of dissection and/or end-organ mal-perfusion syndrome. Beta-blockers and sodium nitroprusside are the most important cornerstone in the management of AD. Verapamil and diltiazem can be used if the patient does not tolerate beta-blockers [8,12].

## Conclusions

Our case demonstrated that the diagnosis of AD can be very challenging. It is important to keep in mind that silent AD can occur rarely and may be an incidental diagnosis. Our patient presented initially with scrotal swelling, and upon further evaluation, CT abdomen revealed a type B AD. The elderly patient had uncontrolled hypertension as well as a history of a recent dizziness followed by fall. Likely the patient had AD-related syncope, orthostatic hypotension or a transient ischemic attack. A high index of suspicion should always be present in high-risk patients like this one. Physicians should always widen their differential diagnosis and look for possible causes of an unexplained dizziness and fall in the elderly hypertensive patient.
